# Morphological growth and carcass performance of crossbred beef × dairy cattle with expression of beef- versus dairy-type

**DOI:** 10.1093/tas/txaf152

**Published:** 2025-11-11

**Authors:** Blake A Foraker, Bradley J Johnson, Ryan J Rathmann, J Chance Brooks, Mark F Miller, Miles E Theurer, Max Garrison, Dale R Woerner

**Affiliations:** Department of Animal and Food Sciences, Texas Tech University, Lubbock, TX 79409, United States; Department of Animal and Food Sciences, Texas Tech University, Lubbock, TX 79409, United States; Department of Animal and Food Sciences, Texas Tech University, Lubbock, TX 79409, United States; Department of Animal and Food Sciences, Texas Tech University, Lubbock, TX 79409, United States; Department of Animal and Food Sciences, Texas Tech University, Lubbock, TX 79409, United States; Veterinary Research and Consulting Services LLC, Hays, KS 67601, United States; Performance Cattle Company LLC, Amarillo, TX 79124, United States; Department of Animal and Food Sciences, Texas Tech University, Lubbock, TX 79409, United States

**Keywords:** conformation, frame score, hip height, muscling, phenotype, ribeye area

## Abstract

Considerable variance in the expression of beef- versus dairy-type among contemporary groups of beef × dairy cattle may contribute to management challenges and financial losses. The objective of this study was to evaluate influence of beef- versus dairy-phenotype expression on growth metrics through the finishing phase and carcass performance. Angus × Holstein or SimAngus × Holstein steers and heifers were managed within 9 contemporary groups through the finishing period and classified at harvest based on frame score and muscling score equally into 1 of 4 phenotype groups (*n* = 82 to 84 cattle per group): (i) fully dairy-type, (ii) partially dairy-type, (iii) partially beef-type, and (iv) fully beef-type. Linear body measurements, carcass ultrasound data, and visual frame and muscling scores were obtained on arrival at the finishing yard and on re-implant. Carcass data were obtained after slaughter. Data were analyzed for effect of phenotype group and its interaction with time during the finishing period. Measurements were compared for their contribution in predicting harvest phenotype outcome. Body weight was not different (*P *= 0.64) between groups, indicating that differences (*P *< 0.05) in muscling and frame scores were not attributable to weight. Fully dairy-type crossbreds had greater (*P *< 0.05) hip height, shoulder height, and body depth than fully beef-type crossbreds at both processing times. Fully beef-type crossbreds had greater (*P *< 0.05) ultrasound 12^th^ rib fat thickness and ultrasound ribeye area than fully dairy-type crossbreds at arrival and re-implant. However, ribeye area at harvest was not different (*P *= 0.32) between phenotype groups, although round muscling score differed (*P *< 0.01) and generally decreased from fully beef-type to fully dairy-type. Ribeye area at harvest was weakly correlated with live animal muscling score (r = 0.15) and round muscling score (*r* = 0.22), whereas live animal muscling was much more highly correlated (r = 0.53) with round muscling. Round muscling was the most indicative of phenotype classification at harvest. Hence, ribeye area at harvest may not be entirely representative of visual phenotype differences in muscling of beef × dairy crossbreds. Longitudinal measurements of carcass traits and frame size may serve as effective sortation tools in minimizing phenotypic variation of beef- and dairy-type among beef × dairy cattle.

## Teaser

Beef × dairy crossbreds with different phenotypes at harvest exhibited morphological differences throughout the finishing phase. Round muscling was much more effective at differentiating muscularity among beef × dairy crossbreds at harvest than ribeye area.

## Introduction

Terms like “appropriate ribeye size”, “uniformity and consistency,” “predominantly black hided,” “structural soundness/conformation,” “moderate frame score”, and “lean to fat ratio” have been used by cattle feeders and beef packers in the United States to describe specified categories of quality in beef production, including lean, fat, and bone, visual characteristics, weight and size, and (or) cattle genetics (Hasty et al. 2017; Igo et al. 2013). Hence, production of beef products from uniform, adequately muscled, and compositionally correct cattle is important for value determination in the beef supply chain. Stark differences in these terms have been used to characterize and appropriately value conventional beef versus dairy cattle and carcasses (Schaefer 2005).

Simultaneous selection for milk production and meat conformation (muscling) in dairy cattle has been suggested to yield little net progress for either milk or meat production systems (Blackmore et al. 1958). Consequently, at equal carcass weights and especially at equal live weights, cattle of dairy breeds are generally less desirable than cattle of beef breeds for quantity of meat production (Adams et al. 1982; Berg and Butterfield 1976; Callow 1961; Griffin et al. 1992). Through computer simulation, the individual cost of an animal being inferiorly muscled versus heavily muscled, which is often the case between dairy and beef cattle, was estimated at approximately $102 in 1995 (Smith et al. 1995). This figure is likely more exaggerated in today’s market because of inflation. Differences in muscling, in part, underpin the disparity in feeder and slaughter cattle prices between conventional beef and Holstein cattle (McKendree et al. 2020; Schulz et al. 2018).

Crossbreeding beef sires with dairy cows has considerably increased the number of crossbred beef × dairy cattle slaughtered in the United States and displaced fed, straightbred dairy slaughter cattle entering the beef supply chain (Baisel and Felix 2022; NAAB 2022). The additive effects of beef breed genetics have been generally thought to result in greater muscling and carcass conformation of beef × dairy versus straightbred dairy progeny (Berry 2021). As a result of their, on average, more beef-type conformation, beef × dairy crossbreds have been valued more closely to conventional beef cattle than straightbred dairy cattle in the feeder cattle market (McCabe et al. 2022).

Several industry leaders, including genetic suppliers, feedlot managers, and beef processors, have noted large variability in visual appearance, namely beef- versus dairy-type, of beef × dairy crossbreds, even within contemporary groups of cattle with similar genetics, management, sex, and age. Some have speculated that beef- versus dairy-type within the beef × dairy crossbred population varies more greatly at harvest than at arrival at the feedlot, suggesting differential growth patterns among the phenotypes. Beef packers often base live pricing on cattle appearance near harvest, not upon arrival. As a result, large variations in expressed beef- versus dairy-type near harvest may negatively affect value. Sorting early in the finishing phase to minimize variation at harvest may present challenges if certain types of beef × dairy crossbreds grow differentially over time.

Most assessments of cattle biological type on feedlot and carcass performance have used cattle breed to define biological type (Camfield et al. 1999; Cundiff et al. 1993; Koch et al. 1976; Nour et al. 1983). Yet, large differences in carcass traits have been suggested within breeds because no single breed excels in all economically relevant traits (Cundiff et al. 1989; Wheeler et al. 1996). Few, if any, studies have considered the influence of biological type on traits of economic importance within cattle of equal breed composition.

Given the importance of uniformity, muscling, and compositional correctness in determining value within the beef supply chain—and the known negative impact of dairy influence on slaughter cattle value—beef × dairy cattle expressing more ideal beef-type characteristics may hold disproportionate value compared to those with stronger dairy-type traits, especially when marketed together. Identification of the most pertinent traits related to beef- versus dairy-type and their changes during finishing may allow for optimal feedlot management of beef × dairy crossbreds. We hypothesized that traits related to muscularity would effectively characterize and sort beef- versus dairy-type among beef × dairy crossbreds throughout the finishing phase. The objective was to evaluate morphological growth and carcass performance among contemporary crossbred beef × dairy cattle that expressed beef- versus dairy-type.

## Materials and methods

Animal handling procedures complied with standards published in the Guide for the Care and Use of Agricultural Animals in Agricultural Research and Teaching (Federation of Animal Science Societies 2020).

### Cattle description

A total of 615 crossbred beef × dairy cattle (arrival body weight: mean = 354 kg, SD = 49.9 kg) were placed at a commercial feedyard in southwest Kansas between July and September 2020. Cattle arrived to the feedyard in 9 shipments (average of 70 cattle each). Cattle within each shipment shared the same sex (6 shipments of steers, 3 shipments of heifers), same calf ranch source, and similar calf management. Birth date records were incomplete. Thus, once cattle were allocated to a pen, an average birth date was calculated from available records for cattle in that pen and used for all individuals in that pen. From available records, cattle within a pen were generally not more than 30 d different in age. When available, sire and dam identity was provided by a commercial genetics company and (or) a recordkeeping system at the originating dairy. Cattle were resulting progeny of Angus or Simmental × Angus sires and a contemporary group of Holstein cows from 3 large commercial dairies (greater than 5,000 cows each) under the same ownership. From the 503 records available for sire identity, it was determined that 3 sires were represented by 50 or more progeny, an additional 5 sires were represented by 20 or more progeny, and an additional 4 sires were represented by 10 or more progeny.

### Cattle processing and management

Cattle were processed at 3 times: (i) arrival, (ii) re-implant, and (iii) harvest. Upon arrival to the feedyard, cattle were provided *ad libitum* access to grass hay and water. Cattle were rested for 12 to 36 h after arrival before processing. Processing at arrival included procedures: (i) placement of a serially numbered tag, (ii) vaccination against bovine respiratory disease and *Leptospirosis sp.* (Titanium^®^ 5 L5 HB; Elanco Animal Health, Greenfield, IN), (iii) vaccination against clostridial disease (Vision^®^ 7 with SPUR^®^; Merck Animal Health, Summit, NJ), and (iv) administration of a trenbolone acetate (100 mg) and estradiol benzoate (14 mg) implant (Synovex^®^ Choice; Zoetis, Parsippany, NJ). After processing, each of 7 shipments (unsorted) were assigned to a pen. To minimize financial losses from differences in weight, steers within 2 additional shipments (same arrival date) were sorted and assigned to 1 of 2 pens by body weight. Thus, a total of 9 pens of cattle were enrolled in the study.

Cattle remained in their assigned pen for the duration of the study, such that cattle within a pen were fed the same diet and endured the same weather events. Cattle were fed twice daily using a slick bunk feeding program, where a trained observer estimated orts to provide near *ad libitum* supply of feed. Diets were formulated to meet or exceed nutritional requirements for feedlot cattle (NASEM 2016; Table 1). Cattle were not fed beta-adrenergic agonists. Cattle were gradually transitioned from diets 1 to 3 depending on consumption, days on feed, and projected body weight. Water was provided *ad libitum* by an automatic float-activated water system. Cattle were processed at an average of 105 d on feed (range: 64 to 120 d on feed; re-implant) for administration of a trenbolone acetate (200 mg) and estradiol benzoate (28 mg) implant (Synovex^®^ Plus; Zoetis, Parsippany, NJ). Cattle were processed for a final time on their date of harvest, which was determined for each pen to target 28% empty body fat using a proprietary endpoint projection system (Performance Cattle Company, LLC, Amarillo TX). Between pens, a total of 592 cattle were harvested at an average of 180 d on feed (range 146 to 213 d on feed). Because cattle within a pen were contemporaries for sex, age, management, source, and harvest endpoint (ie, days on feed), the blocking effect of pen was used to assess study effects of interest (beef- versus dairy-type).

**Table 1.** Ingredient and nutrient composition (dry-matter basis) of rations fed throughout the study.

Item	Ration^a^		
	1	2	3
**Ingredient**			
** Steam flaked corn**	23.7	35.1	59.7
** Wet distillers grain**	30.2	34.2	26.8
** Ground alfalfa hay**	28.8	14.8	6.5
** Fat**	0.0	0.6	1.9
** Corn steep**	14.0	11.6	0.0
** Liquid supplement**	3.3	3.8	5.0
**Nutrient analysis**			
** Dry matter, % as-fed**	69.26	69.54	72.55
** NE_m_, Mcal/kg**	1.74	2.01	2.23
** NE_g_, Mcal/kg**	1.12	1.38	1.57
** Crude protein, %**	17.21	17.50	13.34
** Non-protein nitrogen, %**	2.07	2.34	2.46
** Crude fat, %**	3.17	5.60	7.58
** Crude fiber, %**	17.75	11.11	6.37
** Calcium, %**	1.16	0.92	0.80
** Phosphorus, %**	0.37	0.41	0.36
** Potassium, %**	1.50	1.03	0.66

a Rations for all cattle were formulated to provide 60 to 90 mg·animal^-1^·day^-1^ tylosin (90% DM basis), 26 g/ton monensin, 50 mg·animal^-1^·day^-1^  *Lactobacillus acidophilus* and *Lactobacillus buchneri* (1X 109 CFU·animal^-1^·day^-1^). Rations for heifers also included melengestrol acetate at 0.4 mg·heifer^-1^·day^-1^.

### Cattle measurements

Measurement of body weight was obtained on all cattle at each time of processing (arrival, re-implant, and harvest) using a calibrated scale. No shrink was applied to body weight at arrival and re-implant, but a 4 percent shrink was applied to body weight at harvest. Body weight gain per d on feed (average daily gain) was calculated from arrival to re-implant, re-implant to harvest, and arrival to harvest.

At arrival and re-implant, cattle were carcass ultrasound scanned and imaged at the longissimus thoracis muscle between the 12^th^ and 13^th^ ribs by an experienced technician using an Aloka 500-V real-time ultrasound machine (Corometrics Medical Systems; Wallingford, CT) equipped with a 17.2 cm, 3.5 Mz linear transducer. One ultrasound scan image from each animal at each time point was measured 3 times by the same technician for 12^th^ rib fat thickness and ribeye area, and measurements were averaged.

Linear body measurements taken at arrival and re-implant included hip height, hip length, hip width, 2/3 body length, body depth, and shoulder height. To minimize processing time before shipping and to prevent stress from catching an animal’s head in the squeeze chute, the only linear body measurement taken at harvest was hip height. All linear body measurements were taken by the same experienced technician using the system developed by Performance Cattle Company, LLC (Amarillo, TX). A cable was linked at a clevis between 2 pulleys mounted at opposite ends (front and back) of a squeeze chute at known heights 1 and 2, respectively. Each pulley was attached to a transducer that measured displacement of cable, and the clevis served as the point of reference for measurement. A measurement was recorded in the system by transducers when no cable movement occurred for a constant 0.3 s. Transducers were calibrated to height measurements of 15.2 cm and 106.3 cm before new data collection on each day of processing.

Linear body measurement procedures were similar to those described by Reed et al. (2017) and Kirkpatrick (2020). Upon cattle entrance into the chute, the open end of a “U”-shaped rod of known measurement (height 3) was positioned to transect the animal’s thoracic cavity, where the bottom side of the rod was flush against the front leg and chest floor and where the top side of the rod was perpendicular to the spine. The clevis was positioned directly above the spine and flush with the top side of the rod to capture location 1 and flush with the spine and directly below the top side of the rod to capture location 2. Subsequent measurements were captured with the clevis at a location flush with the spine and medial to the hook bones (location 3), at the distal crest of the left hook bone (tuber coxae; location 4), and at the distal crest of the left pin bone (tuber ischii; location 5). The order of measurements remained the same between animals, and only accurate measurements were recorded (ie, measurements were repeated if an animal moved during the process).

Shoulder height was calculated as the difference between height 1 and the distance between height 1 and location 2. Body depth was calculated as the difference between height 3 and the distance between locations 1 and 2. The distance between locations 2 and 5 represented 2/3 body length. Hip height was calculated as the difference between height 2 and the distance from height 2 to location 3. Hip width was twice the distance between locations 3 and 4. Hip length was the distance between locations 4 and 5.

To reference against visual assessments of frame size, a Beef Improvement Federation (BIF) frame score was calculated using equations provided in BIF (2018), for 5 to 21 mo old bulls (and steers):


$$-11.548+\left(0.19205\times \text{hip}\;\text{height}\;\left[\text{cm}\right]\right)\;–\;\left(0.0289\times \text{age}\;\left[d\right]\right)+\left(0.00001947\times {\;\text{age}}^{2}\right)+\left(0.00001315\times \text{hip}\;\text{height}\times \text{age}\right).$$

and for 5 to 21 mo old heifers:


$$-11.7086+\left(0.1859\times \text{hip}\;\text{height}\;\left[\text{cm}\right]\right)\;–\;\left(0.0289\times \text{age}\;\left[d\right]\right)+\left(0.00001947\times {\;\text{age}}^{2}\right)+\left(0.00002988\times \text{hip}\;\text{height}\times \text{age}\right).$$

### Visual assessments and cattle selection

Visual assessments and cattle selection are described in detail by Foraker et al. (2022). In summary, a panel of 3 expert evaluators independently used a 9-point scale to assess muscling score (1 = light muscled, representative of dairy-type; 9 = heavy muscled, representative of beef-type) and frame size (1 = large frame, representative of dairy-type; 9 = moderate frame, representative of beef-type). Assessments of muscling and frame size for each animal were conducted at arrival, re-implant, and harvest. Scores for muscling and frame size were averaged between panelists and summed to compute a visual phenotype score on a 2- to 18-point scale (2 = extremely dairy-type; 18 = extremely beef-type).

**Fig. 1.**
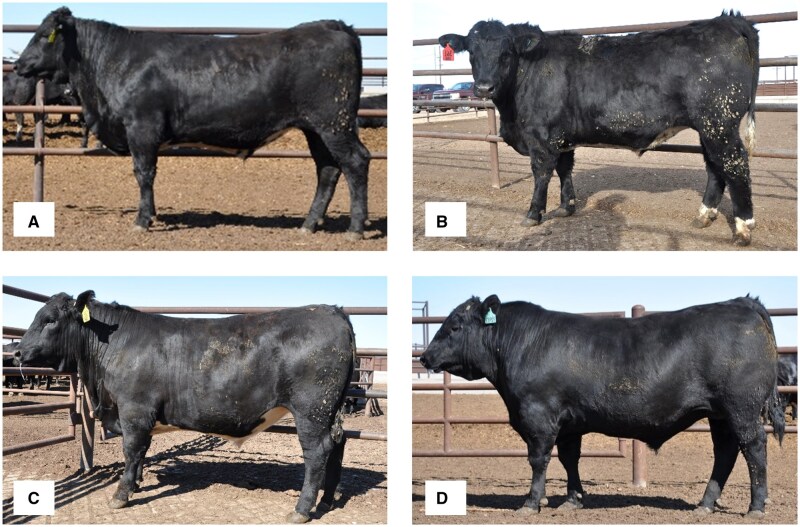
Crossbred beef × dairy steers that represent distinct phenotype groups: (A) fully dairy-type, (B) partially dairy-type, (C) partially beef-type, and (D) fully beef-type.

Using the mean and standard deviation visual phenotype score at harvest within each pen, cattle were categorized into 4 phenotype groups (Fig. 1): (i) fully dairy-type; (ii) partially dairy-type; (iii) partially beef-type; and (iv) fully beef-type. Then, cattle were randomly subset such that each phenotype group within a pen contained an equal number. A total of 333 cattle (*n* = 82 to 84 per phenotype group; *n*_[n]_ = 8 to 11 per phenotype group within each of 9 pens) were selected for inclusion in the study.

The same procedures used to categorize cattle into phenotype groups at harvest were also used to categorize cattle at arrival and re-implant. Classification of cattle at arrival and re-implant into the same phenotype group at harvest using visual phenotype scores was assessed.

### Harvest and carcass measurements

Cattle within a pen were transported either 30 or 300 miles (difference because of plant accessibility for data collection) to a commercial, federally inspected harvest facility and slaughtered on the same day using humane practices according to USDA guidelines. Tag transfer was performed to maintain live animal identity with carcass identity. Livers were scored according to the Eli Lilly Liver Check System (Elanco, Greenfield, IN), and condemnations (only those associated with disease) of all viscera were noted. After final trimming and inspection of carcasses, hot carcass weight was measured using a certified scale, and damage to outside skirt (diaphragm) was recorded. Carcasses were electrically stimulated and then chilled for approximately 30 h. Dressing percentage was calculated as hot carcass weight divided by shrunk body weight at harvest multiplied by 100. Designation of Angus phenotype according to live animal specifications by plant personnel was recorded (USDA-AMS 2017).

After chilling, carcasses were separated between the 12^th^ and 13^th^ ribs, and the exposed lean surface was allowed approximately 20 min to reach an oxygenated state. A portable video image analysis (VIA) system (VBG 2000; e + v Technology GmbH & Co.KG; Oranienburg, Germany) operated by a trained employee was used to capture USDA yield grade and quality grade data from both carcass sides at the exposed lean surface. Grading measurements were averaged between carcass sides, and USDA yield grade and quality grade were calculated (USDA 2017). Carcasses were selected, according to their visual phenotype score and outlined criteria, at the time of grading. The same person evaluated all carcasses for round muscling score: 1 = angular, light muscled, representative of dairy-type; and 9 = bulging, heavy muscled, representative of beef-type. Hindquarter length was measured from the dorsal tip of the aitch bone to the point of 12^th^ and 13^th^ rib separation along the lumbar vertebral column, and forequarter length was measured from the point of 12^th^ and 13^th^ rib separation along the thoracic vertebral column to the juncture of the cervical and thoracic vertebrae. Hindquarter and forequarter lengths were summed to obtain total carcass length.

Carcass weight at arrival and re-implant was estimated using an equation from Tatum et al. (2012): 0.2598 × initial body weight^1.1378^. Using estimated carcass weight at arrival and re-implant and actual carcass weight at harvest, carcass weight gain per day on feed was calculated from arrival to re-implant, re-implant to harvest, and arrival to harvest. Ratio of carcass weight gain to live weight gain was calculated and expressed as a percentage from arrival to re-implant, re-implant to harvest, and arrival to harvest.

### Statistical analyses

Data were analyzed using R statistical software, version 4.1.2 (R Core Team 2021). Significance of effects and pairwise comparisons was established *a priori* at *α *≤ 0.05. Summary statistics (mean and SD) of certain traits for steers and heifers were computed from all cattle (combining those that were selected into phenotype groups and those that were not). Only data measured on selected cattle (experimental unit; *n* = 82 to 84 per phenotype group, stratified across 9 pens) were analyzed.

Data were visually assessed for normality before analysis using boxplots and histograms. For numerical traits (e.g., hip height), the lmer() function from the lme4 package (Bates et al. 2015) was used to fit a restricted maximum likelihood (REML)-based, mixed model. For categorical traits (e.g., liver abscess prevalence), the glmer() function from the lme4 package (Bates et al. 2015) was used to fit a generalized linear mixed model by transforming a binomial response onto a log odds ratio scale. To account for the generalized randomized complete block design of the study, models for all variables were fit with pen (block) and pen × phenotype group interaction as random effects.

Body weight, visual muscling and frame size scores, BIF frame scores, linear body measurements, and ultrasound measurements were modeled with repeated measures (fixed effects: phenotype group, processing time, and phenotype group × processing time interaction; random effects: individual animal identity, pen, and phenotype group × pen interaction). Models for linear body measurements and carcass ultrasound measurements also included covariates (fixed effects) of body weight at each processing time analyzed in the model to adjust measurements to constant body weight for clearer interpretation of results. Carcass measurements (both numerical and categorical variables) were modeled (fixed effects: phenotype group; random effects: pen and phenotype group × pen interaction). Models for numerical carcass measurements included a covariate of body weight at harvest. Live and carcass weight gains at intervals (arrival to re-implant, re-implant to harvest, and arrival to harvest) were modeled (fixed effects: phenotype group; random effects: pen and phenotype group × pen interaction) with initial interval body weight included as a covariate. Correct classification of cattle at arrival and re-implant using visual phenotype scores into their phenotype group at harvest was modeled with repeated measures (fixed effects: phenotype group, processing time, and phenotype group × processing time interaction; random effects: individual animal identity and pen).

Residuals and fitted values from each model were plotted to assess model assumptions for homogeneity of variance. Effects from all models were tested with an analysis of variance (ANOVA) using the anova() function from the lmerTest package (Kuznetsova et al. 2017) with Kenward-Roger approximation of denominator degrees of freedom. When effects were significant, group means were separated with Tukey adjusted pairwise comparisons and Kenward-Roger approximation of denominator degrees of freedom using the emmeans() function from the emmeans package (Lenth 2018).

For each of ribeye area at harvest, round muscling score, and live muscling score at harvest, a linear model was constructed with hot carcass weight as a covariate and pen as a random effect, and residuals of each model were tested for linear relationships. Pearson correlations were calculated using the cor.test() function to evaluate these relationships.

A discriminant function analysis was applied to linear body measurements, carcass ultrasound measurements, and yield related harvest traits. Traits were linearly modeled (random effect: pen), with respective body weight as a covariate (fixed effect). Model residuals were tested for fixed effect of phenotype group using a discriminant function analysis to determine the influence of traits relative to each other in predicting phenotype group at harvest. The candisc package was used to evaluate the canonical correlation of residuals and phenotype group on each discriminant function (Friendly and Fox 2017). Loadings and standardized coefficients were assessed to determine the discriminating ability of each trait.

## Results and discussion

Summary statistics for all crossbred beef × dairy steers and heifers considered for inclusion in this study (not just those that were subset on visual phenotype) are presented in Table 2 to provide context for the sample. After arrival and before harvest, 23 cattle (3.7% of the total) died at the feedyard. From arrival to harvest, steers gained a similar amount of body weight (286 kg) as heifers (283 kg) in 13 d less. Nearly half (46.9 to 48.9%) of cattle (both steers and heifers) contained some level of an abscessed liver condition, and an appreciable proportion (30 to 40%) of cattle exhibited damage to the outside skirt. Carcasses from heifers exhibited an average marbling score of approximately 50 degrees greater than steer carcasses. Consequently, approximately 60% of heifer carcasses exhibited adequate marbling for premium Choice or Prime branded beef programs. Although steer carcasses were 24 kg heavier than heifer carcasses, steers and heifers were largely similar for dressing percentage, fat thickness, ribeye area, and yield grade. As determined by beef processing facility personnel, most steers (97.4%) and heifers (92.3%) expressed sufficient live animal characteristics for eligibility into Angus branded beef programs.

**Table 2.** Summary statistics (unadjusted) for all crossbred beef × dairy cattle (steers and heifers) considered for the study, including those not selected into phenotype groups (N = 333 selected for study).

Item	Steers		Heifers	
	Mean	SD	Mean	SD
**Number of pens**	6		3	
**Number of cattle**	425		190	
**Days of age on arrival**	306	5.3	316	14.1
**Days on feed**				
** Re-implant**	107	21.8	99	26.6
** Harvest**	176	20.7	189	32.3
**Number of cattle deaths**	14		9	
**Body weight** ^a^ **, kg**				
** Arrival**	364	48.9	331	44.1
** Re-implant**	576	47.8	518	49.6
** Harvest**	650	46.2	614	55.9
**Carcass traits**				
** Hot carcass weight, kg**	409	34.0	385	39.7
** Dressing percentage**	62.8	1.72	62.7	2.01
**12th rib fat thickness, cm**	1.34	0.393	1.41	0.395
** Ribeye area, cm** ^b^	85.6	8.29	87.6	9.47
** USDA yield grade**	3.5	0.68	3.3	0.74
** Marbling score** ^b^	473	101.5	520	109.1
**Liver abscess scores**				
** 0, %**	43.1		51.1	
** A, %**	26.1		26.7	
** A+, %**	30.7		22.2	
**Outside skirt damage, %**	40.6		30.3	
**Condemnation of all viscera, %**	25.6		22.1	
**Quality Grade Distribution**				
** Select, %**	18.0		11.0	
** Low Choice, %**	42.6		28.2	
** Upper 2/3 Choice, %**	34.1		50.3	
** Prime**	5.4		10.5	
**Angus phenotype** ^c^	97.4		92.3	

a Body weights reported at arrival and re-implant were not shrunk. Body weights reported at harvest were shrunk 4%. Body weights for animals that died during the study were not removed (deads-in computation).

b 400 = Small 00, 500 = Modest 00.

c Angus phenotype was designated by plant personnel based on American Angus Association requirements for Angus-influence live animal phenotype.

### Visual assessments

From the subset of cattle selected into 4 harvest-determined phenotype groups (fully dairy-type, partially dairy-type, partially beef-type, and fully beef-type), body weight, live muscling scores, and frame size scores determined at 3 processing times are presented in Table 3. Muscling and frame size scores were positively correlated (*r* = 0.57, *P *< 0.01; data not reported) with each other. Yet, inclusion of muscling, frame size, or body weight as covariate(s) did not produce results different from models without covariate(s); hence, analysis of covariance was not used for the results of muscling, frame size, or body weight. Expression of beef- versus dairy-type had no effect (*P *= 0.64) on body weight. Additionally, body weight was not influenced (*P *= 0.98) by a certain phenotype group more than another at different processing times. Ultimately, differences (*P *< 0.01) in muscling and frame size between phenotype groups, irrespective of body weight, indicated that procedures employed in this study to categorize cattle into groups with different morphological body types (beef- versus dairy-type) were effective.

**Table 3.** Body weight, muscling, and frame size at arrival, re-implant, and harvest of crossbred beef × dairy feedlot cattle with different expression of beef- versus dairy-type at harvest.

Item	Fully Dairy-type	Partially Dairy-type	Partially Beef-type	Fully Beef-type	SEM^1^	*P*-value		
						Group	Time	Group × Time
**Number of cattle**	82	84	83	84				
**Body weight** ^2^ **, kg**					10.8	0.64	<0.01	0.98
** Arrival**	354	354	350	353				
** Re-implant**	564	566	557	560				
** Harvest**	642	647	640	642				
**Live muscling score** ^3^					0.21	<0.01	<0.01	0.09
** Arrival**	2.9^a^	3.7^b^	4.3^c^	5.3^d^				
** Re-implant**	3.1^a^	4.0^b^	4.7^c^	5.5^d^				
** Harvest**	2.8^a^	4.0^b^	4.5^c^	5.6^d^				
**Frame size score** ^3^					0.15	<0.01	<0.01	<0.01
** Arrival**	3.4 ^mn^	4.5^o^	5.7^p^	6.5^qr^				
** Re-implant**	3.7^m^	4.7^o^	6.3^q^	7.0^s^				
** Harvest**	3.1^n^	4.4^o^	5.9^pr^	7.1^s^				
**BIF frame score** ^4^					0.17	<0.01	<0.01	0.88
** Arrival**	6.9^a^	6.6^b^	6.0^c^	5.8^c^				
** Re-implant**	7.5^a^	7.1^b^	6.6^c^	6.3^c^				
** Harvest**	7.3^a^	6.9^b^	6.4^c^	6.2^c^				

1 Standard error of the means (SEM), pooled.

2 Body weights reported at arrival and re-implant were not shrunk. Body weights reported at harvest were shrunk 4%.

3 Muscling and frame size were scored by 3 expert evaluators on a scale: 1 = light muscled, large framed, representative of dairy-type; 9 = heavy muscled, moderate framed, representative of beef-type.

4 Beef Improvement Federation (BIF) frame score for 5 to 21 month old bulls (and steers): −11.548 + (0.19205 × hip height [cm])−(0.0289 × age [d]) + (0.00001947 × age^2^) + (0.00001315 × hip height × age), and for 5 to 21 month old heifers: −11.7086 + (0.1859 × hip height [cm])−(0.0289 × age [d]) + (0.00001947 × age^2^) + (0.00002988 × hip height × age).

a-d Estimated marginal means for a trait without a common superscript are different (*P *< 0.05) for phenotype group.

m-s Estimated marginal means for a trait without a common superscript are different (*P *< 0.05) for phenotype group × time.

Differences (*P *< 0.01) between phenotype groups for muscling at harvest also existed at arrival and re-implant. Processing time tended to influence live muscling scores in certain phenotype groups more than others (*P *= 0.09), although this tendency did not seem to have a biologically meaningful effect as phenotype transitioned from dairy-type to beef-type. Processing time by itself, however, was associated (*P *< 0.01) with differences in live muscling scores, which were least (*P *< 0.05) at arrival and not different (*P *> 0.05) between re-implant and harvest. Drennan et al. (2008) also demonstrated that, when the same cattle were evaluated at weaning and slaughter, muscling scores were greater at slaughter than at weaning, which is likely because muscle-to-bone ratio increases as live weight increases (Berg and Butterfield 1976). Live animal estimates of muscling at harvest have been reasonable predictors of carcass conformation and percent meat yield (Drennan et al. 2008; Kauffman et al. 1976; May et al. 2000). Therefore, results here suggested that expression of beef-type muscling in beef × dairy crossbreds may contribute to a greater percent meat yield than expression of dairy-type.

Visual frame size scores were influenced by processing time in certain phenotype groups more than others (*P *< 0.01). Specifically, only cattle in the fully beef-type group were scored smaller (*P *< 0.05) in frame size at both re-implant and harvest than at arrival. The range in mean visual frame size scores across all phenotype groups increased from 3.1 units at arrival to 4.0 units at harvest, a change (nearly 1 unit) which could be considered meaningful on a 9-point scale. This demonstrated that maturing rate was more divergent at time periods closer to harvest than those more distant from harvest. Such a logical growth concept was demonstrated by Fitzhugh and Taylor (1971), where correlations among degrees of maturity increased as age increased. Since visual frame size was defined as skeletal size, these results also suggest that cattle with a greater degree of visual maturity at arrival (like those in the fully beef-type group) were shorter in skeletal size at harvest. This was another theory demonstrated by Fitzhugh and Taylor (1971), although using body weight instead of visual frame size.

Equal emphasis was allocated to muscling and frame size in determining a visual phenotype score and, ultimately, classifying each animal into a phenotype group. The range in mean frame size scores was 30%, 38%, and 43% greater than the range in mean live muscling scores at arrival, re-implant, and harvest, respectively. Hence, evaluators identified that frame size score contributed to a greater amount of variation in visual appearance than muscling. Therefore, measurements of frame size with more objectivity than visual assessment could be beneficial, especially if they account for logarithmic growth.

Calculated BIF frame scores provided a standardized reference to other works. In general, the cattle in this study represented a relatively small range in frame size in comparison to the entirety of the BIF frame score scale. These minimal differences might be expected in cattle of similar breed composition. Even so, differences in frame size, although comparatively small in relation to all cattle, were exploited within the sample to characterize body type differences of beef × dairy crossbreds. Either at similar days of age or at similar body weight to beef × dairy crossbreds in this study, Holstein steers evaluated in Reed et al. (2017) had a BIF frame score of 0.6 to 1.1 units greater than that of fully dairy-type crossbreds and 2.1 to 2.2 units greater than that of fully beef-type crossbreds. Moreover, the standard error of mean BIF frame score in Holsteins of Reed et al. (2017) ranged from 0.07 to 0.09 units, whereas the standard error of mean BIF frame score (not accounting for time and group × time interaction) in this study ranged from 0.10 to 0.12. These results suggested that, despite the influence of beef genetics in moderating frame size of beef × dairy crossbreds compared to Holsteins, as much or more variation in frame size could exist within beef × dairy crossbreds (e.g., beef-type versus dairy-type) as between Holsteins and some beef × dairy crossbreds (e.g., those with fully dairy-type).

### Linear body measurements

Linear body measurements adjusted to constant body weight are presented in Table 4. Hip width was the only linear body measurement influenced (*P *= 0.03) by phenotype group × processing time interaction. At arrival, hip width was not different (*P *> 0.05) between phenotype groups. At re-implant, hip width of fully beef-type crossbreds was 2.8 cm greater (*P *< 0.05) than fully dairy-type crossbreds, signifying a difference in growth of approximately 6%. Hip width at re-implant was numerically intermediate for partially beef- and dairy-type compared to fully beef- and dairy-type. No clear pattern in bone growth has been identified along the vertebral column or appendicular skeleton (Berg and Walters 1983). Although, muscle of the distal limb has been described to develop at a faster rate than muscle at more proximal locations, and fat has shown increasing growth coefficients in a centripetal direction towards the loin (Berg and Walters 1983). Thus, differences in hip width growth were likely less attributable to growth of *tuber coxae* and more attributable to accumulation of muscle and (or) fat at this location. As a result, sortation of beef × dairy crossbreds on hip width at arrival alone may not have meaningful implications at later periods in the finishing phase.

**Table 4.** Linear body measurements adjusted to a constant body weight at arrival (355 kg), re-implant (564 kg), and harvest (644 kg; hip height only) of crossbred beef × dairy feedlot cattle with different expression of beef- versus dairy-type at harvest.

Item	Fully Dairy-type	Partially Dairy-type	Partially Beef-type	Fully Beef-type	SEM^1^	*P*-value		
						Group	Time	Group × Time
**Number of cattle**	82	84	83	84				
**Hip height, cm**					0.68	<0.01	<0.01	0.90
** Arrival**	128.5^a^	127.1^b^	124.4^c^	123.3^d^				
** Re-implant**	138.0^a^	135.9^b^	134.0^c^	132.1^d^				
** Harvest**	140.4^a^	138.2^b^	135.9^c^	134.6^d^				
**Hip width, cm**					0.78	0.31	<0.01	0.03
** Arrival**	60.7^o^	59.9^o^	61.8^o^	59.9^o^				
** Re-implant**	65.2^n^	66.7 ^mn^	66.5 ^mn^	68.0^m^				
**Hip length, cm**					0.67	0.67	<0.01	0.57
** Arrival**	39.9	39.6	39.7	39.2				
** Re-implant**	46.3	46.0	44.7	45.8				
**Shoulder height, cm**					0.66	<0.01	<0.01	0.53
** Arrival**	123.6^a^	121.8^b^	120.2^c^	118.9^c^				
** Re-implant**	130.0^a^	128.2^b^	125.5^c^	124.4^c^				
**2/3 body length, cm**					1.18	0.08	<0.01	0.13
** Arrival**	95.7	93.5	94.5	92.2				
** Re-implant**	107.2	106.0	103.6	104.5				
**Body depth, cm**					1.31	0.04	<0.01	0.13
** Arrival**	59.6^a^	59.1^ab^	59.5^ab^	59.0^b^				
** Re-implant**	72.2^a^	70.6^ab^	69.7^ab^	69.2^b^				

1 Standard error of the means (SEM), pooled.

a,b,c,d Estimated marginal means for a trait without a common superscript are different (*P *< 0.05) for phenotype group.

m,n,o Estimated marginal means for a trait without a common superscript are different (*P *< 0.05) for phenotype group × time.

Across all processing times, hip height increased (*P *< 0.05) in a stepwise fashion from fully beef-type to fully dairy-type. Shoulder height was greatest (*P *< 0.05) in fully dairy-type crossbreds, intermediate (*P *< 0.05) in partially dairy-type crossbreds, and least (*P *< 0.05) in partially and fully beef-type crossbreds, which were not different (*P *> 0.05) from each other. Shoulder height to hip height ratios were not different (*P *= 0.90; data not reported) between phenotype groups, which supports that bone growth, or at least that comprised in hip height and shoulder height measurements, does not occur in a gradient from one body region to another (Berg & Walters 1983).

Fully dairy-type crossbreds exhibited greater (*P *< 0.05) body depth than fully beef-type crossbreds, whereas partially dairy-type and partially beef type were numerically intermediate to fully dairy-type and fully beef-type. Ratio of body depth to shoulder height was influenced (*P *= 0.05; data not reported) by phenotype group, although this ratio tended to be greater (*P *= 0.09) in fully beef-type crossbreds than partially or fully dairy-type crossbreds. Thoracic capacity, which might be related to body depth, has been demonstrated as greater in more angularly shaped than more thickly muscled cattle (Kauffman et al. 1976).

Hip height, shoulder height, and body depth attained at arrival as a percentage of the same measure at re-implant were not different (*P *≥ 0.39) between phenotype groups (data not reported in tabular form). Although some measures indicated differences in maturing rate between phenotype groups, these percentages of growth, like BIF frame scores, suggested that differences in maturity were not drastically different among the phenotype groups in this study. However, the interval in which these measurements were taken (arrival to re-implant) only represented approximately 20% of the average harvest age and a presumably much lesser percentage of the mature age if harvest age was not mature age. Hence, traits measured at one processing time as a percentage of another processing time might be more exaggerated at different stages of life or when a period of greater time is considered.

### Ultrasound measurements

Ultrasound measurements of 12^th^ rib fat thickness and ribeye area at constant body weight are shown in Table 5. Values for 12^th^ rib fat thickness were Box-Cox transformed because variance differed between arrival and re-implant. This demonstrated differences in fat deposition rates among cattle but not necessarily within a particular phenotype group. Since fat deposition follows a logarithmic growth curve, different rates of fat deposition among cattle of different growth types has been demonstrated with ultrasound measures (Berg and Butterfield 1976; Brethour 2001). In this study, rate of fat deposition at the 12^th^ rib between arrival and re-implant was not different (*P *= 0.84) between phenotype groups. Likewise, ribeye area did not (*P *= 0.34) change more in one phenotype group than another between arrival and re-implant. Generally, 12^th^ rib fat thickness increased by 0.43 to 0.46 cm and ribeye area increased by 24 to 27 cm^2^ from arrival to re-implant across all phenotype groups. Averaging over processing times, fully beef-type crossbreds had greater (*P *< 0.05) 12^th^ rib fat thickness than fully dairy-type crossbreds, and fully and partially beef-type crossbreds had greater (*P *< 0.05) ribeye area than fully dairy-type crossbreds. Thus, at any point from arrival to re-implant, fully beef-type crossbreds were inherently fatter and heavier muscled over the 12th rib region than fully dairy-type crossbreds.

**Table 5.** Carcass ultrasound measurements of 12^th^ rib fat thickness and ribeye area adjusted to a constant body weight at arrival (355 kg) and re-implant (564 kg) of crossbred beef × dairy feedlot cattle with different expression of beef- versus dairy-type.

Item	Fully Dairy-type	Partially Dairy-type	Partially Beef-type	Fully Beef-type	SEM^1^	*P*-value		
						Group	Time	Group × Time
**Number of cattle**	82	84	83	84				
**12^th^ rib fat thickness** ^2^ **, cm**					—	0.02	<0.01	0.84
** Arrival**	0.36^b^	0.38^ab^	0.42^ab^	0.44^a^	0.019			
** Re-implant**	0.80^b^	0.84^ab^	0.85^ab^	0.90^a^	0.031			
**Ribeye area, cm** ^2^					1.33	<0.01	<0.01	0.34
** Arrival**	50.4^b^	52.2^ab^	53.6^a^	53.2^a^				
** Re-implant**	74.7^b^	76.0^ab^	78.9^a^	79.9^a^				

1 Standard error of the means (SEM), pooled.

2 Reported values for 12^th^ rib fat thickness were back-transformed from the Box-Cox (lambda = 0.368) scale.

a,b Estimated marginal means for a trait without a common superscript are different (*P *< 0.05) for phenotype group.

As cattle fatten, fat deposition has been shown to increase from the ventral side towards the dorsal side (Belk et al. 1991). Moreover, muscling has been described as developing first at the distal limbs and last in the loin region (Berg and Walters 1983). Similar to this study, the deposition of subcutaneous fat during the finishing period has been shown to occur at an equal rate among feeder cattle varying in frame size and muscle score (Tatum et al. 1986). Thus, at constant age and body weight, ultrasound measurements of muscle and fat at the 12th rib region could be used to hypothesize that fully beef-type crossbreds were more mature at arrival and re-implant than fully dairy-type crossbreds.

### Carcass traits

Analyses of carcass traits at a constant hot carcass weight did not alter interpretation of results from analyses at a constant body weight; thus, analyses of carcass traits were conducted at a constant body weight for consistency with analyses of cattle measurements (Table 6).

**Table 6.** Carcass traits (adjusted to a constant body weight at harvest of 644 kg) of crossbred beef × dairy cattle with different expression of beef- versus dairy-type.

Item	Fully Dairy-type	Partially Dairy-type	Partially Beef-type	Fully Beef-type	SEM^1^	*P*-value
**Number of cattle**	82	84	83	84		
**Dressing percentage** ^2^	62.7^b^	62.7^b^	62.9^ab^	63.5^a^	0.25	0.01
**Carcass traits**						
** Hot carcass weight, kg**	404.7^b^	403.9^b^	405.5^ab^	409.5^a^	1.47	<0.01
** 12th rib fat thickness, cm**	1.30	1.39	1.33	1.47	0.056	0.07
** Ribeye area, cm** ^2^	85.5	87.1	87.8	87.2	1.34	0.32
** USDA yield grade**	3.43	3.43	3.45	3.55	0.08	0.24
** Marbling score** ^3^	487	480	492	493	18.6	0.81
** Round muscling score** ^4^	3.9^c^	4.4^b^	4.8^ab^	5.3^a^	0.19	<0.01
** Carcass length, cm**	154.0^a^	152.2^b^	150.5^c^	149.1^d^	0.41	<0.01
** Hindquarter length, cm**	85.5^a^	84.7^a^	83.7^b^	83.0^b^	0.32	<0.01
** Forequarter length, cm**	68.4^a^	67.5^a^	66.9^c^	66.2^d^	0.27	<0.01
**Liver score**						
** 0, %**	40.6	45.6	48.7	55.4	8.0	0.45
** A, %**	29.5	25.4	27.4	15.9	5.38	0.20
** A+, %**	26.8	26.9	21.4	27.2	6.65	0.83
**Outside skirt damage, %**	42.9	42.4	30.1	33.3	6.69	0.39
**Condemnation of all viscera, %**	27.6	25.2	15.8	19.2	5.73	0.26
**USDA quality grade**						
** Select, %**	13.5	11.8	9.5	9.6	5.99	0.85
** Low Choice, %**	35.9	38.6	37.9	36.2	6.70	0.98
** Upper 2/3 Choice, %**	35.7	38.5	37.5	39.8	7.39	0.96

1 Standard error of the means (SEM), pooled.

2 Dressing percentage was not adjusted to constant body weight because of its part-whole relationship.

3 Marbling scores: 400 to 499 = Small, 500 to 599 = Modest.

4 Round muscling score: 1 = light muscled, representative of dairy-type; 9 = heavy muscled, representative of beef-type.

a-d Estimated marginal means within a row without a common superscript are different (*P *< 0.05).

Ribeye area was not different (*P *= 0.32) between phenotype groups. This result was perplexing because of the group differences in ultrasound measurement of ribeye area at arrival and re-implant. Lack of differences in ribeye area might be explained by maturity rate. Beef-type beef × dairy crossbreds may have ascertained a more mature ribeye size at re-implant than dairy-type beef × dairy crossbreds, thereby slowing subsequent growth in ribeye size until harvest. Contrarily, dairy-type beef × dairy crossbreds may have continued to mature in the later-developing region of the loin through the finishing phase until harvest. This result at harvest might suggest an interaction between maturing rate and ribeye area, especially at the end of the finishing phase.

Phenotype groups were not different (*P *= 0.07) in 12^th^ rib fat thickness at harvest. Although ultrasound was used at arrival and re-implant to theoretically predict rather than definitively measure 12^th^ rib fat thickness, it is noteworthy that the standard error of mean 12^th^ rib fat thickness increased at each measurement time and was greatest at harvest (nearly double from re-implant). This further emphasizes variability in 12^th^ rib fat thickness increases as days on feed increases. Still, 12^th^ rib fat thickness at harvest was numerically greater, by 0.17 cm, in fully beef-type crossbreds than in dairy-type crossbreds; these same groups were only 0.10 cm different (in the same direction) at re-implant. This result provided further evidence that greater variance, rather than lack of effect size, contributed to the insignificant result of 12^th^ rib fat thickness at harvest. When slaughtered at a constant days on feed, cattle of more moderate frame size have been shown to contain greater subcutaneous carcass fat (not just that at the 12^th^ rib) than cattle of larger frame size (Tatum et al. 1986).

Combined, results of ribeye area and 12^th^ rib fat thickness tell a story of subtle differences in maturing rates, where fully beef-type crossbreds were physiologically more mature at harvest than fully dairy-type crossbreds. As maturity increases, it has been recognized that muscle develops at a decreasing rate, while fat develops at an increasing rate (Berg and Butterfield 1976; Owens et al. 1995). Hence, when cattle of different maturing rates are harvested at a constant age, ribeye area at arrival or re-implant may be a relatively poor predictor of ribeye area at harvest. This study demonstrated that, at harvest, fully beef-type crossbreds reached a point of diminishing growth in ribeye area and began to deposit fat at a greater rate than fully dairy-type crossbreds. Harvesting dairy-type crossbreds at a later age would likely increase fat deposition, while the expected change in ribeye size cannot be determined from these data. Body composition has been suggested as constant when measured at a given level of maturity (Owens et al. 1995). Ribeye area and 12^th^ rib fat thickness have been indicative of body composition as employed in the USDA yield grade equation; however, this has been debated, especially in Holstein cattle (Lawrence et al. 2010). Although USDA yield grade was not different (*P *= 0.24), given other indications of subtle differences in maturing rates between phenotype groups, beef-type crossbreds might produce similar carcass composition to dairy-type crossbreds when harvested at an earlier age.

Fully beef-type crossbreds yielded greater (*P *< 0.05) hot carcass weight, greater (*P *< 0.05) round muscling, and lesser (*P *< 0.05) carcass length than either partially or fully dairy-type crossbreds. Further, fully dairy-type crossbreds were least (*P *< 0.05) in round muscling and greatest (*P *< 0.05) in carcass length. These results provide further affirmation that differences in the dispersion of live animal weight created visual phenotype differences attributable to carcass length and width.

Kauffman et al. (1976) demonstrated greater carcass weights were produced when live body weight and live muscling score increased but carcass weights were not influenced by carcass fatness. The authors showed an inverse relationship between thoracic capacity and dressing percentage. Therefore, because cattle in this study were harvested and compared at constant body weight, the greater carcass weights of beef-type crossbreds might be attributed to greater muscularity and lesser thoracic capacity. This result was consistent with live animal measurements if body depth was any predictor of thoracic capacity. Because of their deficiency in muscle, dairy-type crossbreds may be able to attain similar carcass weights to beef-type crossbreds when harvested at heavier body weights, but perhaps at a detriment to carcass composition. Although it may be less influential than muscling on hot carcass weight, carcass fatness cannot be disregarded for its contribution to total carcass composition. Kauffman et al. (1976) showed nearly no relationship between dressing percentage and carcass composition in the form of percentage lipid-free muscle.

**Fig. 2.**
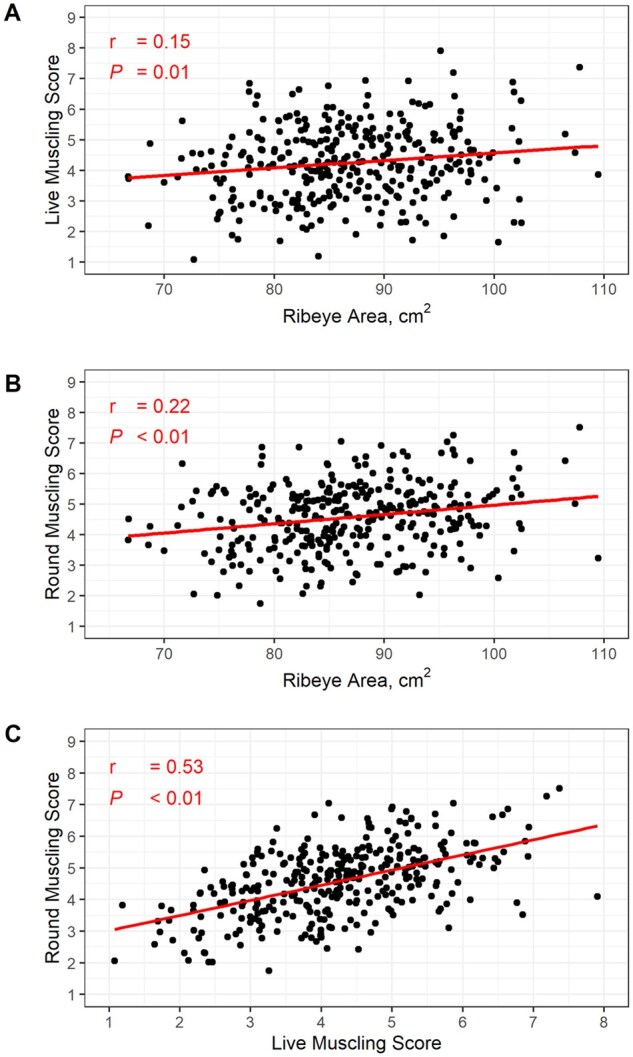
Relationships between ribeye area, live muscling score, and round muscling score (after accounting for pen effects and adjusting to constant hot carcass weight) of crossbred beef × dairy cattle (*N* = 333).

The difference between phenotype groups in round muscling but not ribeye area was puzzling. For clarity, interpretation of these results was not altered when groups were compared at constant 12^th^ rib fat thickness (data not reported). To further explore this phenomenon, relationships between ribeye area, live muscling score, and round muscling score (after accounting for pen effects and adjusting to constant hot carcass weight) were evaluated (Fig. 2). These relationships showed that ribeye area was not a more reasonable predictor of round muscling score (*r* = 0.22, *P *< 0.01) than live muscling score (*r* = 0.15, *P *= 0.01). Rather, live muscling score and round muscling were much more strongly related (*r* = 0.53, *P *< 0.01) and directionally indicative of each other. If live muscling score is truly representative of retail product yield like previously suggested (Drennan et al. 2008; Kauffman et al. 1976; May et al. 2000), then these data would indicate that ribeye area is a comparatively poorer indicator of retail product yield than either live muscling or round muscling scores.

Distribution of carcass muscling has long been demonstrated as constant among muscles, with no difference attributed to wide variations in cattle breed (Berg et al. 1978). According to this principle, dairy-type crossbreds should contain the same proportion of muscle in any region as beef-type crossbreds, although in a more lengthwise, rather than widthwise, capacity. If they had distributed loin muscle in a more lengthwise direction, dairy-type crossbreds should have exhibited less loin muscle width (and ribeye area, indirectly) than beef-type crossbreds. Round muscling scores were reflective of bulge and thickness; intuitively, greater round muscling scores represented weight in a shorter and thicker dimension, and vice versa. Not only does the apparent disconnect between ribeye area and round muscling demonstrate implications on total carcass muscularity, but it implies that ribeye area may be a relatively poor predictor of muscling within the loin itself. In other words, ribeye area may not reflect the alleged constancy in distribution of weight between the round and loin muscles. This hypothesis should be inferred under context that round muscling score was a somewhat subjective measurement.

No differences (*P *> 0.05) existed between phenotype groups for liver abscess scores, outside skirt damage, and visceral condemnations. Numerically, however, the prevalence of liver abscesses was approximately 15 percentage units less in fully beef-type crossbreds than fully dairy-type crossbreds (*P *= 0.45). Partially and fully beef-type crossbreds also experienced 10 to 12 percentage units less (numerically) outside skirt damage than partially or fully dairy-type crossbreds, which was likely related to their lower liver abscess prevalence. Cattle with liver abscesses, and especially those with severe liver abscesses, have exhibited lower dressing percentages than cattle with no liver abscesses (Brown and Lawrence 2010). The lower dressing percentage in dairy-type beef × dairy crossbreds could also be partially attributed to their greater outside skirt damage because hot carcass weight was measured after outside skirts were trimmed. Liver size and visceral size has not been extensively studied, especially as they relate to abscessed liver conditions. However, it could be hypothesized that greater body depth (and presumably greater thoracic capacity) developed in dairy-type beef × dairy crossbreds to accommodate a larger liver and (or) viscera.

Marbling score and quality grade distributions were not different (*P *≥ 0.81) between phenotype groups. Marbling has been described as a later developing depot than other fat depots, and increased time on feed has produced concomitant increases in marbling (Kempster 1981; Miller et al. 1987). However, Bruns et al. (2004) proposed that marbling develops with a plateau (or quadratic) response over time but a linear response over growth (carcass weight). Since phenotype groups were selected to differ in their physiological maturity, it could be expected that earlier-maturing groups deposit marbling at a faster rate than later-maturing groups. If our data aligned to the theory by Bruns et al. (2004), it appears that all phenotype groups had attained a nearly maximal (or close to it) level of marbling at harvest. Likely due to the extended days on feed in this study, differences in maturing rates between phenotype groups had minimal effect on marbling. It remains whether marbling between phenotype groups would be similar at earlier harvest times and fewer days on feed.

### Weight gain

Weight gains were calculated on both a live weight and carcass weight basis to understand carcass tissue accretion between phenotype groups at processing time intervals (Table 7). Gains for an interval were adjusted to a constant initial weight of that interval to remove variation attributable to previous interval gain. While body weights were measured at each processing time, carcass weight was only measured at harvest. Carcass weights at arrival and re-implant were strictly estimations using an equation from Tatum et al. (2012).

**Table 7.** Weight gains adjusted to a constant initial interval weight^1^ during finishing of crossbred beef × dairy feedlot cattle with different expression of beef- versus dairy-type.

Item	Fully Dairy-type	Partially Dairy-type	Partially Beef-type	Fully Beef-type	SEM^2^	*P*-value
**Number of cattle**	82	84	83	84		
**Body weight gain, kg/d**						
** Arrival to re-implant**	2.01	2.02	1.99	1.98	0.058	0.62
** Re-implant to harvest**	1.02	1.06	1.08	1.09	0.058	0.32
** Arrival to harvest**	1.59	1.62	1.60	1.60	0.041	0.85
**Carcass weight gain** ^3^ **, kg/d**						
** Arrival to re-implant**	1.38	1.39	1.37	1.36	0.039	0.63
** Re-implant to harvest**	0.68^b^	0.69^ab^	0.73^ab^	0.77^a^	0.049	0.03
** Arrival to harvest**	1.09	1.10	1.10	1.12	0.027	0.60
**Carcass: live weight gain, %**						
** Arrival to re-implant**	68.7	68.7	68.7	68.7	0.13	0.71
** Re-implant to harvest**	66.5^ab^	64.8^b^	68.9^ab^	72.4^a^	2.43	0.04
** Arrival to harvest**	68.2^b^	68.0^b^	68.8^ab^	70.0^a^	0.57	<0.01

1 Weight gains from arrival to re-implant and from arrival to harvest were adjusted to a constant body weight at arrival of 355 kg. Weight gains from re-implant to harvest were adjusted to a constant body weight at re-implant of 564 kg.

2 Standard error of the means (SEM), pooled.

3 Carcass weight at arrival and re-implant was estimated using an equation from Tatum et al. (2012): 0.2598 × initial body weight^1.1378^.

a,b Estimated marginal means within a row without a common superscript are different (*P *< 0.05).

Rate of body weight gain (otherwise, average daily gain) was not different (*P *≥ 0.32) between groups for any interval during the finishing phase. Carcass weight gains were not different (*P *= 0.63) from arrival to re-implant but were greater (*P *< 0.05) in fully beef-type crossbreds than fully dairy-type crossbreds from re-implant to harvest. Still, this difference did not seem to impact overall carcass weight gain, which was not different (*P *= 0.60) from arrival to harvest. This result suggested dressing percentage at harvest was not different enough between phenotype groups to impact total carcass gain, if the groups exhibited equal dressing percentages at arrival.

Carcass to live weight gain (otherwise, carcass transfer) was not different (*P *= 0.71) from arrival to re-implant but was greater (*P *< 0.05) from re-implant to harvest in fully beef-type crossbreds than in partially dairy-type crossbreds. For the entire feeding period (arrival to harvest), carcass transfer was greater (*P *< 0.05) in fully beef-type crossbreds than in partially or fully dairy type crossbreds. The digestive tract has been suggested to reach its mature size and weight before other carcass components (Owens et al. 1995). As live weight nears maturity, live growth rate begins to slow, whereas carcass growth rate remains linear (Streeter et al. 2012). Because of the discrepancy on whether this weight might be fat (Owens et al. 1995) or muscle (Kauffman et al. 1976), a serial slaughter evaluating phenotype expression in beef × dairy crossbreds would need conducted to determine an ideal harvest endpoint for the population.

### Phenotype misclassification

Because sorting and (or) harvesting phenotype groups at different time points could have implications on cattle value, the ability of evaluators to sort cattle at arrival or re-implant into their same phenotype group at harvest was assessed. Calculated probabilities for correct classification at arrival and re-implant are shown in Table 8. Generally, when not correctly classified into their phenotype group at harvest, cattle were classified into a phenotype group not more than one grouping level different from their correct phenotype group. At chance, the probability of a correct classification in this study was 0.25. All probabilities of correct classification at arrival and re-implant were greater than 0.50. The probability of correctly classifying a certain phenotype group at one processing time more than another at a separate processing time was not different (*P *= 0.15). Correct classification was also not altered (*P *= 0.75) by phenotype group alone. However, the probability of correct classification was 15 percentage units greater (*P *< 0.05) at re-implant than arrival. This result suggested that visual appearance at harvest was more equal to appearance at re-implant than appearance at arrival, which follows logic of temporality. Correct classification by visual assessment of cattle into the harvest-determined phenotype groups was 50 to 70% between arrival and re-implant and presumably less at earlier times. Because managing and marketing crossbreds with beef-type at harvest separately from those with dairy-type may have financial implications, additional sorting tools that are more objective than visual assessment might be effective in making such distinctions.

**Table 8.** Probability of a 3-member panel to classify (using muscling and frame size scores) crossbred beef × dairy cattle into the same phenotype group at arrival and re-implant as the final phenotype group determined at harvest.

	Phenotype Group at Harvest							
Item	Fully Dairy-type	Partially Dairy-type	Partially Beef-type	Fully Beef-type	SEM^1^	*P*-value		
						Group	Time	Group × Time
**Number of cattle**	82	84	83	84				
**Probability** ^2^					0.06731	0.75	0.05	0.15
** Arrival**	0.515^b^	0.529^b^	0.522^b^	0.618^b^				
** Re-implant**	0.674^a^	0.559^a^	0.709^a^	0.568^a^				

1 Standard error of the means (SEM), pooled.

2 Probability values were back-transformed from the logit scale. Tests were performed on the log odds ratio scale.

a,b Estimated marginal means for a trait without a common superscript are different (*P *< 0.05) for time.

### Relationships between traits and phenotype groups

**Fig. 3.**
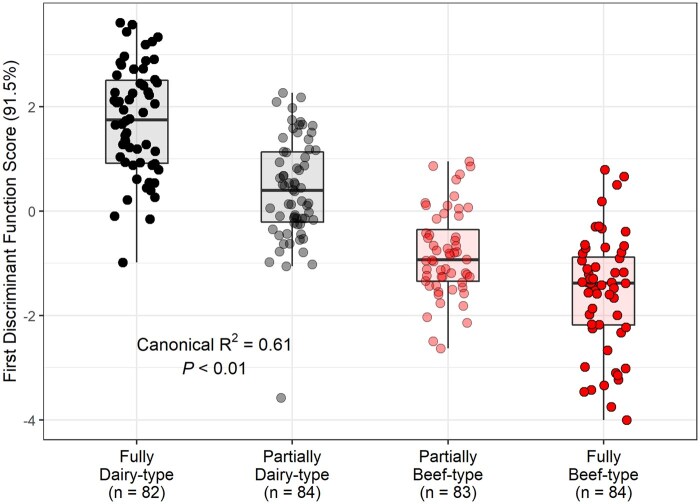
Relationships between ribeye area, live muscling score, and round muscling score (after accounting for pen effects and adjusting to constant hot carcass weight) of crossbred beef × dairy cattle (*N* = 333).

Sorting tools were compared to each other for their potential effectiveness in predicting visually determined harvest outcomes. A discriminant function analysis was conducted to predict visually determined phenotype group at harvest with supplementary traits (adjusted for constant body weight at respective measurement time and pen effects) measured at arrival, re-implant, and (or) harvest. Phenotype group prediction from the first linear combination (discriminant function), calculated with all traits, is shown in Fig. 3. Only the first discriminant function separated (*P *< 0.01) phenotype groups, so the remaining 2 functions (*P *> 0.89) were not assessed. This function explained over 90% of the variability between traits and predicted 61% of the variability in these traits between phenotype groups. From predicted scores using the first function, all phenotype groups were different (*P *< 0.01) from each other. However, partially and fully beef-type groups were more similar to each other than any other pair of groups.

Loadings showed the relationship between a trait and the function, and standardized coefficients demonstrated relative contribution of one trait to another (Table 9). In essence, loadings demonstrated what has already been discussed—the relationship of phenotype group to a trait. Only standardized coefficients were a primary interest here, so that one trait could be assessed for its ability to predict phenotype group compared to other traits. Without exception, round muscling at harvest was most predictive of phenotype group classification and warranted some measure of hindquarter muscling might be important to classification at earlier processing times. Hip width at arrival and re-implant seemed to be relatively unimportant to phenotype group classification and was not a strong predictor of hindquarter muscling. Hip height at each processing time, as well as shoulder height at arrival, was comparatively important to discerning phenotype groups. This result aligns with the concept that bone is an earlier maturing tissue than muscle or fat. Thus, bone should remain relatively proportional to growth, at least more so than other body tissues. When other traits contributed to phenotype group at harvest, they were contributive at earlier rather than later processing times. Ultrasound measurements of ribeye area and 12^th^ rib fat thickness at arrival were stronger predictors of phenotype group than the same measures at re-implant or harvest, which supports previous discussion of these traits. Body depth and 2/3 body length at arrival had intermediate contributions to phenotype group classification.

**Table 9.** Loadings and standardized coefficients^a^ on the first discriminant function of objective traits^2^ at 3 processing times (a = arrival, RI = re-implant, H = harvest) of crossbred beef × dairy cattle (*N* = 333).

Item	Loadings	Standardized coefficients
***Objective trait***		
** Hip height—A**	0.61	0.27
** Hip height—RI**	0.67	0.24
** Hip height—H**	0.73	0.34
** Hip width—A**	0.02	0.10
** Hip width—RI**	−0.19	−0.04
** Hip length—A**	0.09	−0.01
** Hip length—RI**	0.10	−0.01
** 2/3 body length—A**	0.21	0.22
** 2/3 body length—RI**	0.26	0.08
** Body depth—A**	0.11	−0.20
** Body depth—RI**	0.20	0.08
** Shoulder height—A**	0.56	0.37
** Shoulder height—RI**	0.59	0.14
** Ultrasound ribeye area—A**	−0.24	−0.24
** Ultrasound ribeye area—RI**	−0.30	−0.07
** Ribeye area—H**	−0.12	0.11
** Ultrasound 12^th^ rib fat thickness—A**	−0.32	−0.25
** Ultrasound 12^th^ rib fat thickness—RI**	−0.22	0.13
** 12^th^ rib fat thickness—H**	−0.13	0.05
** Round muscling score—H**	−0.63	−0.55
***Canonical R^2^***	0.61	
***Eigen Value***	1.56	
***P-Value (H_0_: canonical R^2^ = 0)***	<0.01	

a Loading indicates the relationship between a variable and discriminant function, and standardized coefficient indicates unique contribution of a variable to a function.

These results suggested that determination of phenotype group membership at harvest is possible by using some objective tools early in the finishing phase. Thus, potential exists to sort cattle into harvest groups using these techniques. Use of some traits, like those associated with fat and muscle, as sorting criteria may be more effective at earlier, rather than later, times in the finishing phase when cattle differ in maturing rates. Measurements more closely associated with bone growth seem to be less affected by time interval. Consideration should be given to objective measurements that can characterize musculature of the hindquarter earlier in the finishing phase because round muscling was a primary determinant of beef- versus dairy-type at harvest.

## Conclusions

Differences in morphological body type (particularly width in relation to height) were demonstrated between beef × dairy crossbreds exhibiting beef- versus dairy-type. A disconnect between ribeye area and live muscling scores, even at constant weight, indicated a need for additional, or at least more accurate, measures beyond ribeye area to assess total animal muscling. Round muscling was demonstrated as a key differentiator in beef- versus dairy-type, so technologies aimed at quantifying carcass yield and total animal muscularity may be best suited focusing on this anatomical region. Sorting techniques employed early in the feeding phase, particularly if focused on measures of skeletal frame, hindquarter muscularity, and maturing rate, may effectively reduce variation in beef- versus dairy-type at harvest. It remains uncertain how differences in beef- versus dairy-type of beef × dairy crossbreds at harvest may directly relate to feedlot profitability and carcass fabrication yields.
